# Ménétrier-like disease in a dog without glandular atrophy: expanding the morphologic spectrum

**DOI:** 10.1186/s12917-026-05424-9

**Published:** 2026-03-25

**Authors:** Mario Pultrone, Dyana Erba, Margherita Orlandi, Michela Pugliese

**Affiliations:** 1 Clinica Veterinaria Checkup Vet, Salerno, 84131 Italy; 2 Anicura Policlinico Veterinario Roma Sud, Rome, 00173 Italy; 3 Private Veterinary Laboratory - MyLav, Milan, 20017 Italy; 4 Department of Veterinary Sciences, University of Messina, Messina, 98168 Italy

**Keywords:** Protein-losing enteropathy, Canine gastrointestinal disease, Gastric mucosal hypertrophy

## Abstract

**Background:**

Ménétrier’s disease is a rare protein-losing gastropathy characterized by markedly enlarged gastric folds, excessive mucus secretion, and hypoalbuminemia. Histologically, it features pronounced foveolar hyperplasia, cystic dilation of gastric glands, and loss of parietal and chief cells. Although first described in humans, a small number of comparable cases have been reported in dogs, where the condition is referred to as *Ménétrier-like disease*. In veterinary medicine, this disorder remains poorly defined and appears to be uncommon.

**Case presentation:**

A 6-year-old intact male French Bulldog was referred for a severe progressive weight loss and acute onset anorexia. Clinical and laboratory findings were suggestive of a protein-losing enteropathy. Abdominal ultrasonography revealed marked thickening of the gastric and small intestinal walls. Gastroscopy showed severe, non-reducible hypertrophy of the gastric folds involving both the fundus and body of stomach. Endoscopic biopsies revealed marked foveolar hyperplasia and cystic glandular dilation without evidence of glandular atrophy. Ménétrier-like hypertrophic gastropathy was considered the most likely diagnosis. Given that the owner declined gastrectomy and treatment with somatostatin analogues for financial constraints, medical management with glucocorticoids and H₂-receptor antagonists was administered. After 10 days, the clinical condition progressively worsened, with the onset of new and evolving neurological deficits. The dog subsequently developed severe neurological signs, and euthanasia was ultimately elected.

Histopathological examination of a full-thickness gastric sample confirmed mucosal hypertrophy characterized by branching foveolar hyperplasia and cystic dilation of the glands, again without evidence of parietal cell loss.

**Conclusions:**

This case suggests that canine Ménétrier-like disease may occur even in the absence of glandular atrophy, thereby broadening the currently recognized histologic spectrum of the condition.

## Introduction

Ménétrier’s disease is a rare protein-losing gastropathy described in humans, clinically characterized by hypoalbuminemia and, histologically, by giant hypertrophic gastric folds associated with excessive mucus production [1]. From a microscopic standpoint, the condition is defined by marked foveolar hyperplasia, cystic dilation of the gastric glands, and loss of parietal and chief cells consistent with glandular atrophy [2]. Although originally recognized in humans, a limited number of canine cases sharing comparable clinical and histopathological features have been reported, leading to the term “Ménétrier-like disease” in dogs [3–8]. In veterinary medicine, this condition remains poorly characterized and appears to be uncommon. Diagnostic criteria have not been standardized, and most cases are identified based on endoscopic and histopathological similarities to the human syndrome [4–6, 8]. In dogs, however, glandular atrophy is inconsistently described and may even be absent, raising the question of whether it should be considered an essential diagnostic criterion in the veterinary setting [4–6, 8]. This case report describes a French bulldog with severe hypertrophic gastropathy. This was confirmed by endoscopic evaluation and full-thickness histopathology. The dog fulfilled the morphological criteria for Ménétrier-like disease, except for glandular atrophy. This case further enhances our understanding of the disease spectrum and highlights the need for a broader diagnostic approach to Ménétrier-like disease in canine patients.

## Case presentation

A 6-year-old intact male French Bulldog was referred for a three-month history of progressive weight loss, chronic vomiting, and worsening anorexia. On the physical examination, the dog appeared cachectic and lethargic, with hypothermia and a body condition score of 1/9 [9]. Feces were reported as normal; no clinical signs suggestive of intestinal involvement were reported. The dog was being fed a commercial monoprotein dry diet based on pork. Hematological and biochemical analyses were performed; results and corresponding reference intervals are summarized in Table 1. Complete blood count revealed moderate normocytic, normochromic, non-regenerative anemia, moderate thrombocytopenia, and mild lymphopenia. Serum biochemistry showed hypoproteinemia, hypoalbuminemia, azotemia, hyperphosphatemia, and increased hepatic transaminases (ALT 107 U/L; AST 91 U/L). Creatinine concentration was within the normal range. Urinalysis was unremarkable, with no evidence of proteinuria or abnormalities suggestive of renal protein loss. Gastrointestinal function testing revealed a trypsin-like immunoreactivity (TLI) value > 50 μg/L, folate > 24 μg/L. Cobalamin and cPL level and basal cortisol concentration were within normal limits. Serological tests for *Leishmania infantum*, *Ehrlichia* spp., *Anaplasma* spp., *Borrelia burgdorferi*, and *Dirofilaria immitis* were negative.

**Table 1:** Hematological and biochemical abnormalities observed in a French Bulldog diagnosed with Ménétrier-like disease

Parameter	Result	Reference interval
Packed cell volume (%)	26.8	37–55
Red blood cells (M/μL)	3.66	5.5–8.5
Reticulocytes (K/μL)	10.6	11–92
Platelets (K/μL)	76	160–430
Lymphocytes (K/μL)	0.82	1.0–4.8
Total proteins (g/dL)	4.3	5.7–8.3
Albumin (g/dL)	2.1	2.5–3.6
BUN (mg/dL)	100.5	19.0–50.0
BUN/Crea Ratio	117.64	10–20
Phosphorus (mg/dL)	5.13	2.5–5.0
ALT (U/L)	107	0–75
AST (U/L)	91	0–52
GGT (U/L)	13	0–12
TLI (μg/L)	> 50	5.2–35
Folate (μg/L)	> 24	7.7–24

**Fig. 1:**
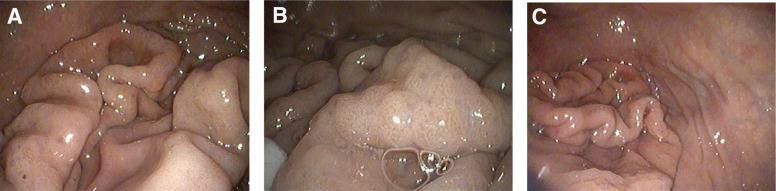
Gastroscopic examination of the stomach. **A** Marked hypertrophy of the gastric rugae; (**B**) Cerebriform appearance of the gastric mucosa; (**C**) Non-reducible gastric folds upon air insufflation

**Fig. 2:**
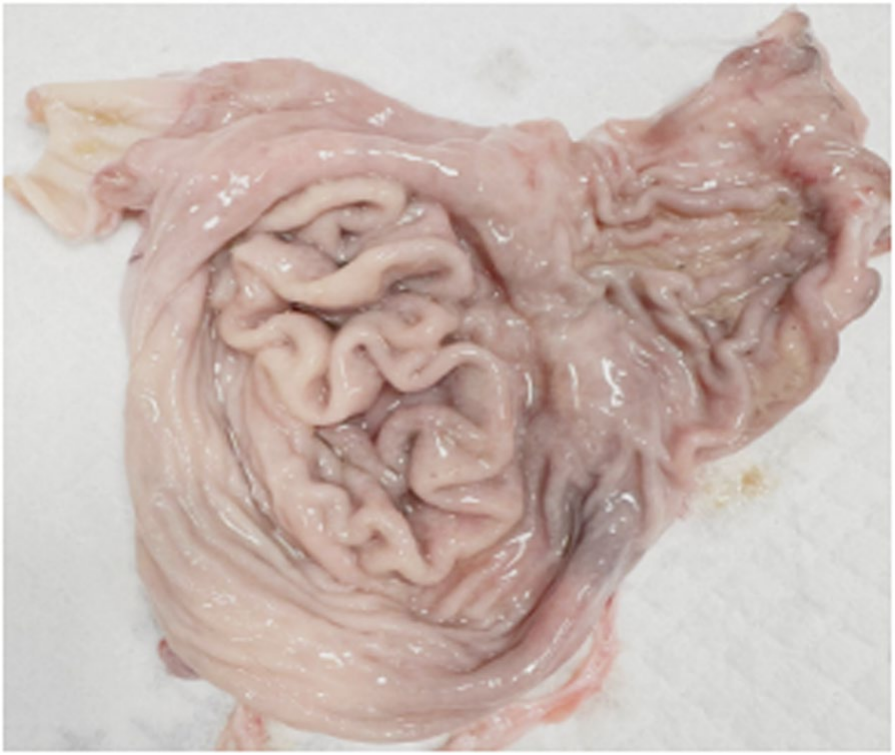
Necropsy image depicting diffuse hypertrophy of the gastric rugae and a cerebriform mucosal architecture. The rugal folds remain non-pliable and fail to flatten when manually stretched

**Fig. 3:**
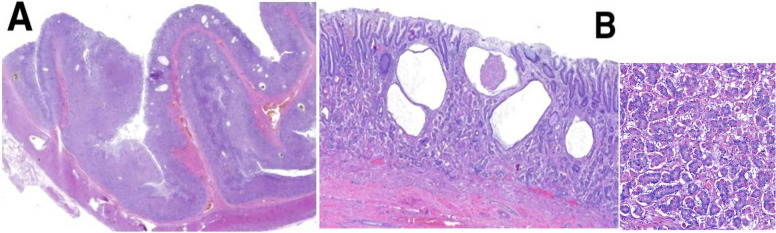
Full-thickness histological sections of gastric fundus in a dog with Ménétrier-like disease (H&E stain). **A** The gastric mucosa showed a marked and diffuse hyperplasia with prominent folds (40x). **B** Hyperplasia and branching of the gastric pits, associated with multifocal cystic dilatation of the glandular structures due to proteinaceous secretion, and multifocal surface erosion (inset: presence of gastric glands lined by both parietal and chief cells) (200x)

Abdominal ultrasonography showed diffuse thickening of the gastric wall with preservation of normal wall layering. An endoscopy and a thoraco-abdominal CT scan were proposed, but the owner declined the latter due to financial constraints. Gastroscopy revealed severe gastric mucosal abnormalities characterized by marked rugal hypertrophy, resulting in a cerebriform appearance, with folds that remained non-reducible upon air insufflation (Fig. 1A–C). The lesions predominantly affected the fundus and body of the stomach, whereas the pyloric antrum appeared endoscopically spared. The mucosa was diffusely hyperreflective and erythematous. Multiple endoscopic biopsies were obtained from both the affected gastric regions and the duodenum, fixed in a 10% buffered formalin solution, and submitted for histopathological examination. Biopsies were routinely processed and embedded in paraffin; 4 µm sections were stained with hematoxylin and eosin (H&E). Histopathological evaluation of the gastric mucosa revealed pronounced foveolar hyperplasia with papillary projections and cystic glandular dilatation. The lamina propria was expanded by oedema and moderate fibrosis, associated with a lymphoplasmacytic infiltrate. Duodenal biopsies showed villous blunting, lamina propria fibrosis, and mild lymphoplasmacytic infiltration. These findings were suggestive of Ménétrier-like disease, although an infiltrative neoplastic process involving the deeper gastric layers could not be excluded based solely on endoscopic biopsies. At this stage, a presumptive diagnosis of Ménétrier-like disease was made, supported by the histopathological features, clinical signs, hematobiochemical abnormalities, ultrasonographic changes, and endoscopic findings. Medical treatment was initiated immediately after the endoscopy with oral prednisone at 1 mg/kg once daily and ranitidine at 2 mg/kg twice daily and continued following histologic evaluation. Adjunctive treatment with octreotide was proposed but declined because of cost considerations, as was subtotal gastrectomy. The patient showed only mild and transient clinical improvement. Ten days after diagnosis, the dog was presented for an acute onset of neurological signs, including obtundation, disorientation, and vestibular symptoms, such as head tilt and ataxia, raising suspicion of a cerebrovascular event. Due to the rapid deterioration and poor prognosis, the owners elected compassionate euthanasia. The entire stomach was submitted for post-mortem examination (Fig. 2). Full-thickness histopathology subsequently confirmed diffuse mucosal hypertrophy, extensive foveolar hyperplasia, and marked cystic glandular dilatation, with no parietal cell atrophy or neoplastic transformation (Fig. 3 A-B). To the authors' knowledge, this represents one of the rare veterinary cases in which the complete stomach was available for histological assessment, allowing definitive exclusion of neoplasia and confirmation of the absence of glandular atrophy. The final diagnosis was consistent with a Ménétrier-like disease, exhibiting the hallmark epithelial and glandular hyperplasia, but lacking the classical feature of glandular atrophy typically described in the human counterpart.

## Discussion

Ménétrier disease is a rare protein-losing gastropathy in humans, histologically characterized by marked foveolar hyperplasia, cystic glandular dilation, and glandular atrophy, typically resulting in hypoalbuminemia and gastric enlargement [1, 2]. Since its original description, sporadic cases in dogs and, more recently, in cats with similar clinicopathological features have been reported, leading to the designation “Ménétrier-like disease” in veterinary medicine [3–8, 10, 11]. However, in contrast to the human syndrome, glandular atrophy in animals is inconsistently documented or absent, raising important questions about its diagnostic value across species [4, 5, 7, 8]. The present case shares many clinicopathological features with those observed in both human and canine reports, including severe hypoalbuminemia, vomiting, weight loss, ultrasonographic evidence of gastric mucosal thickening with preserved layering, and characteristic endoscopic appearance of giant, non-reducible rugal folds [4, 5, 8]. The marked cachexia observed in this patient was interpreted as predominantly attributable to advanced gastric protein loss. In human Ménétrier’s disease, as well as in previously reported veterinary cases, weight loss has been primarily ascribed to protein-losing gastropathy compounded by reduced nutritional intake secondary to chronic vomiting [1, 2, 4]. Although mild duodenal alterations were identified, no histologic evidence of primary intestinal protein-losing enteropathy was documented. While a minor intestinal contribution cannot be entirely excluded, the overall clinical, biochemical, and histopathologic findings strongly support a predominantly gastric origin of protein loss and the consequent cachectic state. The elevated urea-to-creatinine ratio recorded in this case was considered most consistent with a prerenal mechanism, likely secondary to dehydration associated with chronic vomiting and inadequate intake. No clinical, laboratory, or post-mortem findings supported primary renal disease. This interpretation informed supportive management, which focused on fluid correction and hemodynamic stabilization [12].

The non-regenerative anemia observed was likely multifactorial, potentially reflecting anemia of chronic inflammation, occult gastrointestinal blood loss from mucosal erosions, and nutritional deficiencies related to prolonged protein depletion [13]. The normocytic, normochromic indices are consistent with a chronic disease process and further support the presence of a longstanding systemic disorder in the context of advanced hypertrophic gastropathy [4].

Endoscopic mucosal biopsies revealed foveolar hyperplasia and cystic dilatation, fulfilling key diagnostic criteria. However, the recognized limitation of endoscopic sampling in assessing deeper mucosal and submucosal layers must be acknowledged [2]. Accordingly, infiltrative processes such as lymphoma, adenoma, or carcinoma could not be definitively excluded at that stage [5–7].

To the authors’ knowledge, this is among the few veterinary cases in which the entire stomach was submitted for post-mortem histologic examination, enabling robust exclusion of neoplasia and confirmation of Ménétrier-like disease despite the absence of glandular atrophy [2–4, 8]. Of the ten veterinary reports reviewed, only a minority included full-thickness or organ-wide sampling [3, 5, 6]. In most cases, the diagnosis relied on partial sampling (endoscopic or surgical), which limits the ability to exclude focal neoplastic transformation. The absence of glandular atrophy in the described case aligns with previous reports suggesting that this feature may not be essential for the diagnosis in dogs [3, 4, 7, 8]. This has significant implications for case recognition, especially in early or atypical presentations.

Medical treatment with prednisone was initiated based on the suspected inflammatory component of the gastropathy and supported by prior reports of glucocorticoid-responsive cases [4, 14]. Octreotide, a somatostatin analogue that has shown benefit in human Ménétrier disease by downregulating TGF-α/EGFR signaling, was considered as a rational adjunctive therapy but declined for financial reasons [15, 16], as was subtotal gastrectomy. Although some canine cases have shown clinical improvement following partial gastrectomy [4, 17], surgical intervention may not be feasible in all patients because of anatomic, clinical, or owner-related factors. In contrast to previously reported veterinary cases in which clinical improvement was achieved following glucocorticoid administration or partial gastrectomy [4, 14], the present patient exhibited only a minimal and transient therapeutic response. In human disease, therapeutic outcomes are heterogeneous and appear influenced by disease chronicity and the extent of mucosal remodeling [13]. The marked hypoalbuminemia and severe cachexia documented here likely reflect an advanced disease stage, which may account for the limited therapeutic response. Variability in clinical course and outcome should therefore be regarded as part of the spectrum of canine Ménétrier-like disease. The development of acute neurological signs within 10 days of diagnosis precluded long-term evaluation of treatment efficacy. A suspected cerebrovascular event ultimately led to euthanasia. Similar rapid systemic deterioration has been described in at least one prior veterinary report [6, 8]. Whether severe hypoalbuminemia or an unrecognized concurrent condition contributed to the neurologic manifestations remains speculative.

From a diagnostic standpoint, this case emphasizes that in the presence of marked gastric fold hypertrophy, profound hypoalbuminemia, and highly characteristic and concordant endoscopic findings, the absence of glandular atrophy on mucosal biopsies should not automatically be interpreted as sampling error or indirect evidence of occult malignancy. In this patient, histologic findings from endoscopic biopsies were fully concordant with those observed at comprehensive post-mortem examination. This clinicopathologic coherence supports the reliability of in vivo diagnosis when imaging, biochemical, endoscopic, and histologic findings demonstrate internal consistency. In carefully selected cases, a high degree of diagnostic confidence may therefore be achieved even in the absence of demonstrable glandular atrophy. An association between Ménétrier’s disease and *Helicobacter pylori* has been described in human medicine; however, a definitive cause–effect relationship remains controversial and has not been consistently demonstrated across studies [2, 15].

In human medicine, an association between Ménétrier disease and *Helicobacter pylori* infection has been described, although a definitive causal relationship remains controversial [2, 15]. In veterinary patients, gastric colonization by *Helicobacter* spp. is relatively common and not invariably associated with overt disease [18, 19]. Nonetheless, concurrent infection has been reported in isolated cases of Ménétrier-like gastropathy [7], suggesting a possible contributory or modulatory role in susceptible individuals. In the present case, no spiral organisms were identified on routine histopathology, and no ancillary testing (special stains, immunohistochemistry, or molecular assays) was performed. Consequently, while there is no direct evidence of infection, an etiologic role for *Helicobacter* spp. cannot be definitively confirmed or excluded.

Limitations of this report include the absence of pre-mortem advanced imaging (CT) and the lack of immunohistochemical staining to further characterize the epithelial and inflammatory phenotype. Nonetheless, the availability of full-thickness, organ-wide histology strengthens the reliability of the diagnosis and allows us to assert with confidence the absence of glandular atrophy or neoplasia. The concurrent mild duodenal changes are of uncertain significance but may reflect protein-losing enteropathy of mixed gastric-intestinal origin.

## Conclusion

In conclusion, this case contributes to the veterinary literature by showing that canine Ménétrier-like disease may occur without glandular atrophy. It highlights how marked foveolar hyperplasia and cystic glandular changes, when combined with characteristic clinical, imaging, and endoscopic findings, may suffice for diagnosis — particularly in patients not previously treated with proton pump inhibitors. The striking concordance observed in this patient between endoscopic and post-mortem histology suggests that in vivo diagnosis might be achievable with reasonable confidence in appropriately selected cases and by experienced clinicians. Although not pursued here, treatments such as octreotide or partial gastrectomy have been associated with clinical benefit in the literature.

## Data Availability

All data generated or analyzed during are included in this article.
